# Transcriptomic and Polysomnographic Insights Into Core Signaling Pathways in Obstructive Sleep Apnea

**DOI:** 10.1155/mi/4579543

**Published:** 2025-09-28

**Authors:** Peijun Liu, Xiaolan Yang, Guang cai Li

**Affiliations:** ^1^Department of Respiratory and Critical Care Medicine, The Central Hospital of Enshi Tujia and Miao Autonomous, Enshi, China; ^2^Department of Respiratory and Critical Care Medicine, Renmin Hospital of Wuhan University, Wuhan, China; ^3^Department of Pediatrics, The Central Hospital of Enshi Tujia and Miao Autonomous Prefecture, Enshi, China

## Abstract

**Background:** This study integrates transcriptomics and polysomnography (PSG) to investigate the core signaling pathways underlying obstructive sleep apnea (OSA), aiming to elucidate its complex pathophysiological mechanisms. These findings may provide new perspectives on the prevention, diagnosis, and treatment of OSA.

**Methods:** Participants underwent PSG to monitor indicators, such as total sleep time, apnea–hypopnea index (AHI), oxygen desaturation index (ODI) and lowest oxygen saturation (LSO_2_). Individuals with AHI > 5 were categorised into the OSA group, while others were classified as the regular snoring group. Total RNA from white blood cells was extracted using the TRIzol method, and transcriptomic data were obtained via high-throughput sequencing. Weighted Gene Coexpression Network Analysis (WGCNA) and Protein–Protein Interaction (PPI) network analysis were used to identify OSA-related core genes. Differential gene expression, Gene Ontology (GO) and Kyoto Encyclopedia of Genes and Genomes (KEGG) enrichment analyses were conducted to explore key signaling pathways. Single-cell sequencing validated the findings, and Mendelian randomization (MR) analysis confirmed causal links between genes and pathways.

**Results:** While no significant differences were observed between the OSA and regular snoring groups in gender, age, or body mass index (BMI), significant disparities were noted in sleep parameters such as AHI, ODI, and LSO_2_. Principal component analysis (PCA) revealed transcriptomic differences between the groups. WGCNA identified 302 differentially expressed genes (DEGs), with the Palevioletred module significantly correlating with PSG parameters. GO and KEGG analyses implicated core genes in regulating inflammation, viral defence, cell growth, and apoptosis, highlighting the NF-κB signaling pathway as central to OSA pathogenesis. PPI analysis identified key genes, including CEBPB and SPI1, while single-cell sequencing suggested NF-κB pathway activation affecting T cell subgroup distribution. MR confirmed causal relationships between core genes and the NF-κB pathway.

**Conclusion:** This study identified significant differences in sleep parameters between OSA and regular snoring groups and revealed core genes enriched in the NF-κB signaling pathway. These findings suggest that targeting the NF-κB pathway may offer therapeutic benefits for OSA.

## 1. Introduction

Obstructive sleep apnea (OSA) affects nearly 1 billion individuals globally and has garnered increasing attention since the early 21st century [1]. OSA is characterized by repeated partial or complete upper airway obstructions during sleep, resulting in chronic intermittent hypoxia (CIH) and sleep disruption [2]. It is also associated with excessive daytime sleepiness, adversely impacting quality of life and health outcomes [3]. Polysomnography (PSG) remains the gold standard for diagnosing OSA, providing comprehensive assessments of sleep physiology [4]. PSG continuously records various physiological signals, including respiration, electroencephalogram (EEG), electromyogram (EMG), and electrocardiogram (ECG), throughout the sleep cycle. This enables the derivation of critical parameters such as the apnea–hypopnea index (AHI), peak respiratory rate (PRR), mean apnea–hypopnea duration (MAHD), longest apnea duration (LAD), number of apnea–hypopnea events (NAHE), mean saturation of oxygen (MSO_2_), lowest saturation of oxygen (LSO_2_), and oxygen desaturation index (ODI) [5–7]. These metrics are essential for evaluating sleep quality and assessing OSA severity.

Bioinformatics, which integrates computational technology, statistics and biology, has become an indispensable tool in analyzing complex medical datasets [8]. This study leverages PSG and transcriptomic data from individuals with OSA and snoring to investigate the genetic underpinnings of OSA. By analyzing these datasets, we aim to identify key signaling pathways and critical genes involved in OSA pathogenesis. The findings are intended to advance understanding of OSA's molecular mechanisms and inform the development of targeted diagnostic and therapeutic strategies.

## 2. Materials and Methods

### 2.1. Study Population

This study included patients presenting with nighttime snoring who were admitted to the Department of Respiratory Medicine at Renmin Hospital of Wuhan University between May and August 2022. Upon admission, participants underwent routine evaluations, including complete blood count, lipid profile analysis, and liver and kidney function assessments. Within 1 week of the initial evaluation, overnight PSG was performed to assess sleep patterns and episodes of apnea.

Patient diagnosis and classification followed the guidelines for the diagnosis and treatment of OSA [9]. Based on their AHI scores, participants were divided into two groups: those with an AHI of less than 5 events/h were assigned to the non-OSA (non-OSA) group, indicating that while they exhibited snoring symptoms, they did not meet the criteria for an OSA diagnosis. Conversely, participants with an AHI greater than 5 events/h were classified as having OSA. All participants provided informed consent, and the study protocol was approved by the Ethics Committee of Renmin Hospital of Wuhan University.

### 2.2. Inclusion Criteria

The inclusion criteria for the study aimed to ensure a broad and diverse participant pool, with particular attention to high-risk populations. Eligible participants were required to be between 18 and 70 years old, ensuring a wide range of the adult population. A body mass index (BMI) of 18–40 kg/m^2^ ensured that individuals ranging from normal weight to various levels of obesity were included, allowing for representation across different weight categories. Additionally, participants were required to either self-report symptoms of snoring or have snoring reported by family members.

### 2.3. Exclusion Criteria

Participants were excluded if they had any of the following conditions: severe respiratory diseases, such as Grade II–IV chronic obstructive pulmonary disease or pulmonary embolism; significant cardiovascular diseases, including unstable angina or recent myocardial infarction; severe mental disorders that could interfere with data analysis; long-term use of sleep medications or drug dependence that could alter natural sleep patterns; neurological conditions affecting the central regulation of breathing; recent facial or upper airway surgeries that could impact respiratory function; recent acute respiratory infections; or severe obesity (BMI > 40 kg/m^2^), which could hinder the accurate assessment of respiratory function.

### 2.4. Comprehensive Sleep Monitoring With PSG

PSG was employed to record detailed sleep parameters. Sensors and electrodes placed on the head and face measured brain activity, eye movements, and muscle activity. Nasal airflow sensors, thoracoabdominal belts and pulse oximeters recorded airflow, respiratory effort, and oxygen saturation. Key metrics included: AHI, PRR, MAHD, LAD, NAHE, MSO2, LSO2, and ODI.

### 2.5. Sample Collection Protocol

Whole blood samples were collected from both groups in sterile, RNase-free tubes to ensure RNA integrity and prevent degradation. Approximately 5 mL of blood was layered over an equal volume (5 mL) of Ficoll density gradient medium in 15 mL centrifuge tubes (1:1 ratio). The samples were centrifuged at 800 rpm for 20 min at 4°C. The white blood cell layer at the plasma-Ficoll interface was carefully extracted using a pipette and transferred to fresh tubes. The cells were then diluted with PBS, centrifuged at 300 rpm for 5 min, washed, and resuspended for further analysis.

### 2.6. Total RNA Extraction

Total RNA was extracted from the collected white blood cells using the TRIzol method. A volume of 4 mL of RNAiso Plus was added to the cells for lysis, followed by incubation for 5 min to ensure complete cell disruption. Subsequently, 800 μL of chloroform was added, and the mixture was shaken vigorously for 15 s and left to stand for 3 min. The mixture was centrifuged at 12,000 rpm for 15 min at 4°C, and RNA from the aqueous phase was carefully transferred to a fresh tube. The RNA was then precipitated by adding 500 μL of isopropanol, incubated for 10 min,and pelleted by centrifugation at 12,000 rpm for 10 min. The RNA pellet was washed with 1 mL of 75% ethanol, gently mixed, and centrifuged at 7500 rpm for 5 min. After removing the ethanol, the pellet was air-dried, dissolved in 50 μL of DEPC-treated water, and stored at −80°C for subsequent analyses.

### 2.7. Transcriptome Data Processing and Analysis

Raw sequencing data were processed using Trimmomatic to remove low-quality reads and adapter sequences. Deduplication was performed using Unique Molecular Identifier (UMI) technology to generate consensus sequences. Reads were aligned to the reference genome using STAR, and expression levels were quantified using featureCounts with RPKM (Reads Per Kilobase per Million mapped reads) normalization. Differential expression analysis was performed using EdgeR, employing dispersion estimation and *p*-value adjustment to identify significantly differentially expressed genes (DEGs).

### 2.8. Gene Set Enrichment Analysis (GSEA)

GSEA was conducted using GSEA software (v4.1.0) to analyze genome-wide expression data. Genes were ranked within predefined gene sets, and enrichment scores were calculated to determine the degree of clustering of gene sets at the top or bottom of the ranked list. Higher enrichment scores indicated stronger associations with specific biological pathways or states. Random control distributions were used to calculate *p*-values, identifying gene sets significantly linked to the observed biological conditions.

### 2.9. Identification of Gene Modules Associated With OSA Clinical Features

Weighted Gene Co-expression Network Analysis (WGCNA) was employed to identify gene modules associated with clinical parameters of OSA, such as the AHI, LSO_2_ and ODI. WGCNA constructed a coexpression network by calculating Pearson correlation coefficients among genes and determining an optimal soft threshold to define connection strengths. A soft-thresholding power of 16 was selected as it was the lowest power at which the scale-free topology fit index reached a value above 0.85, indicating a biologically meaningful network structure. Hierarchical clustering grouped genes with similar expression patterns into modules, which were then analyzed for associations with OSA-related clinical features. Key modules with potential biological relevance to OSA pathophysiology were identified for further exploration.

### 2.10. GO and KEGG Enrichment Analysis

Gene Ontology (GO) enrichment analysis was performed on intersecting genes using the TopGO package (version 2.42.0) in Bioconductor. The analysis covered three domains: biological process (BP), molecular function (MF) and cellular component (CC), providing comprehensive insights into gene functional classifications and underlying mechanisms. Kyoto Encyclopedia of Genes and Genomes (KEGG) enrichment analysis identified significant gene enrichment in signaling pathways, uncovering their biological implications. The Benjamini–Hochberg method was applied to correct for multiple testing, effectively controlling the false discovery rate and ensuring the robustness of the results.

### 2.11. Protein–Protein Interaction (PPI) Network Analysis and Core Gene Identification

PPI data for DEGs were retrieved from the STRING database (https://string-db.org/) to construct a PPI network. The network was visualized using Cytoscape software (version 3.8.0) with layout algorithms to highlight key modules and nodes. Topological features, such as degree and betweenness, were used to evaluate node importance. Core genes within the PPI network were identified through this analysis using Cytoscape's built-in plugin tools.

### 2.12. Immune Infiltration Analysis

The CIBERSORT algorithm was used to analyze gene expression data by estimating the relative abundance of different immune cells in each sample based on their unique gene expression signature sets. Immune cell types were selected using a significance threshold of *p*  < 0.05. The Volcano Plot package was employed to create violin plots illustrating immune cell infiltration patterns.

### 2.13. Single-Cell Sequencing Analysis

Single-cell sequencing data from the GEO database (GSE214865), comprising samples from the University of Missouri Sleep Clinic and representing diverse racial groups and BMI ranges, were analyzed [10]. Using Seurat v5.0.1, low-quality data were filtered, UMI counts normalized, and log-transformed. Highly variable genes were identified, and dimensionality reduction and clustering were performed using principal component analysis (PCA) and UMAP. Cluster marker genes were identified using the Wilcoxon rank-sum test (adjusted *p*  < 0.01, log fold-change ≥ 0.25). GSEA (KEGG database) and AUCell were used to assess NF-κB pathway activation in T cells, calculating AUC scores based on gene expression. Results were visualized on UMAP plots using ggplot2.

### 2.14. Mendelian Randomization(MR) Analysis

Two-sample MR analysis was conducted using data from GWAS databases. Single nucleotide polymorphisms (SNPs) associated with core genes were selected as instrumental variables (IVs). Two GWAS datasets were analyzed: one related to core gene expression and the other to associated signaling pathways. MR analysis was performed using the TwoSampleMR package on the MR-Base platform. Causal relationships were evaluated using methods such as inverse variance weighting (IVW) and MR-Egger regression. Sensitivity and heterogeneity analyses ensured the robustness and reliability of the findings.

### 2.15. Statistical Analysis

Statistical analyses were performed using SPSS 25.0. The Shapiro–Wilk test was used to assess the normality of continuous variables. Normally distributed data were expressed as mean ± SD and analyzed using the *t*-test. Nonnormally distributed data were expressed as median (Q_1_, Q_3_) and analyzed using the Mann–Whitney *U* test. Categorical data were presented as *n* (%) and compared using Fisher's exact test. A *p*-value < 0.05 was considered statistically significant.

## 3. Results

### 3.1. Comparative Analysis of Clinical Data Between the OSA and Control Groups

A total of 28 participants were initially enrolled in the PSG monitoring project. Four participants withdrew due to discomfort, and RNA samples from six participants degraded, resulting in 18 participants being included in the final analysis. The cohort comprised nine patients diagnosed with OSA and nine patients with primary snoring. No significant differences were observed between the OSA and snoring groups in terms of sex, age, or BMI (*p*  > 0.05). However, key metrics such as AHI, LAD, NAHE, MSO2, LSO2, and ODI showed significant differences between the groups (*p*  < 0.05) (Table 1).

### 3.2. PCA of Transcriptomic Data

**Figure 1:**
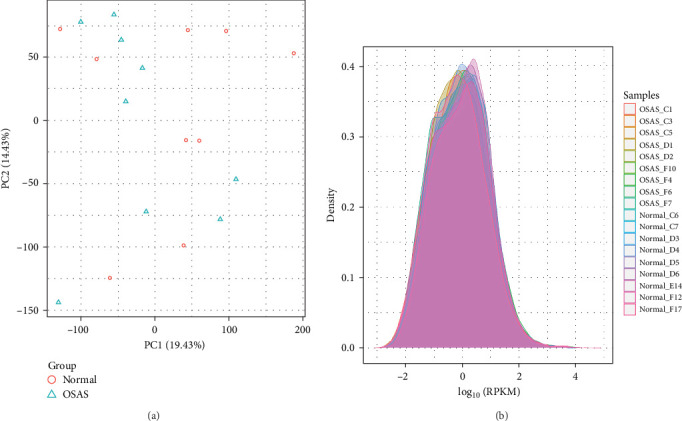
Principal Component Analysis of OSA and Control Groups. (A) PCA plot. (B) Gene expression density plot.

PCA was performed to reduce the dimensionality of the transcriptomic data. The PCA results indicated partial separation between the two groups along PC1 and PC2. PC1 accounted for 19.43% of the total variance, and PC2 accounted for 14.43%. Samples from the OSA group primarily clustered in the lower-left region of the PCA plot, whereas control group samples were dispersed in the upper region (Figure 1A). The density distribution plot of gene expression levels displayed curves representing the density of gene expression for each sample, expressed as log-transformed RPKM values, with different-colored curves corresponding to individual samples (Figure 1B).

### 3.3. WGCNA Analysis Based on Transcriptomic and PSG Data

**Figure 2:**
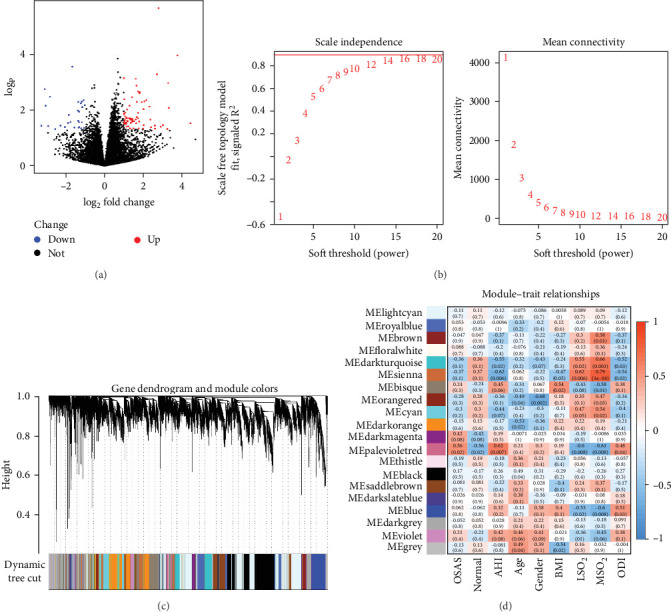
Integrated analysis of transcriptomic and PSG data. (A) Volcano plot of differentially expressed genes. (B) Soft-threshold power plot for WGCNA. (C**)** Gene module clustering dendrogram in WGCNA. (D) Heatmap of gene module correlations with PSG data. Module numbers indicate the correlation coefficients between gene modules and PSG parameters, with numbers in parentheses representing statistical *p*-values.

To identify biomarkers and elucidate the mechanisms of OSA, WGCNA was applied to analyze gene coexpression and its association with PSG data. A total of 302 DEGs were identified using the criteria |log_2_FC| > 1 and *p*  < 0.05 (Figure 2A). In the WGCNA analysis, a soft threshold power of 16 was determined to yield the most stable gene coexpression network (Figure 2B). Gene clustering revealed distinct modules based on expression patterns, with hierarchical clustering producing a gene tree segmented at different heights to define specific modules (Figure 2C). The Palevioletred module exhibited significant correlations with OSA pathogenesis and PSG parameters, particularly AHI. Within this module, 199 genes demonstrated significant correlation coefficients of 0.62 with AHI, 0.6 with LSO_2_ and 0.61 with MSO_2_, all statistically significant (*p*  < 0.05) (Figure 2D).

### 3.4. Screening and Enrichment Analysis of Core Genes in OSA

**Figure 3:**
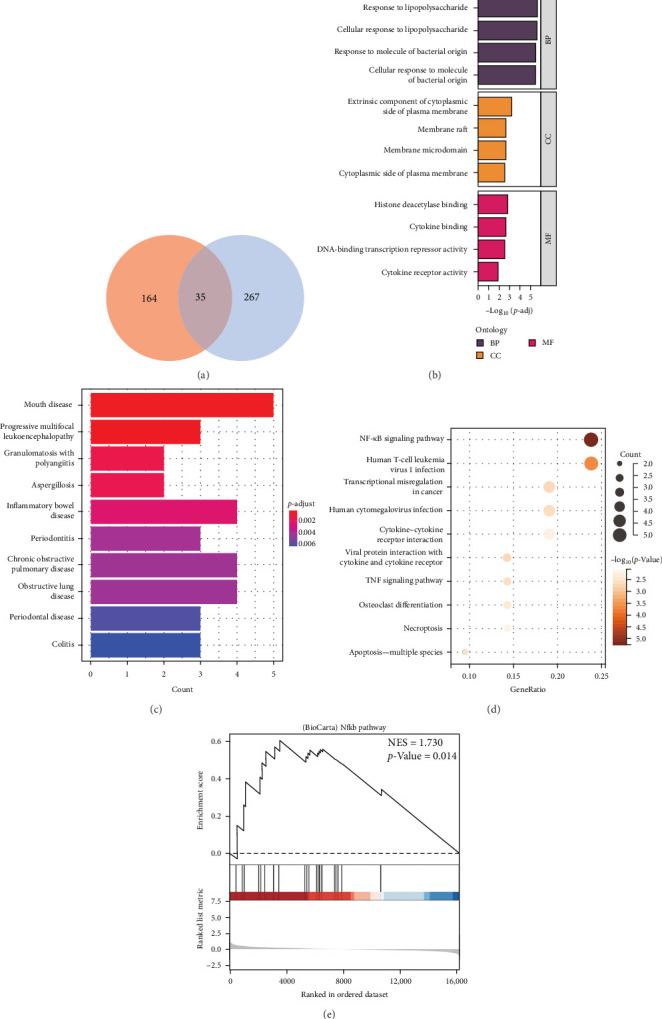
Selection and enrichment analysis of key genes in OSA. (A) Venn diagram showing the intersection of differentially expressed genes and palevioletred module genes. (B) GO enrichment analysis of key genes, highlighting their roles in biological processes, cellular components, and molecular functions. (C) DO enrichment analysis linking key genes to diseases, such as oral disorders and pulmonary heart disease. (D) KEGG pathway analysis showing enrichment in pathways including NF-κB signaling, human cytomegalovirus infection, and apoptosis. (E) GSEA validating the enrichment of the NF-κB signaling pathway.

Intersection analysis of the Palevioletred module genes identified through WGCNA with DEGs yielded 35 key genes significantly associated with clinical indicators, such as AHI and ODI (Figure 3A). GO enrichment revealed their roles in inflammation and immune regulation, particularly through BPs like “response to lipopolysaccharide” and “response to bacterial molecules.” CCs such as “membrane raft” and “cytoplasmic side of the plasma membrane” highlight their involvement in membrane-related signaling, while MFs like “histone deacetylase binding” and “cytokine binding” suggest transcriptional regulatory activities (Figure 3B). DO enrichment linked these genes to diseases like “oral disorders,” “pulmonary heart disease,” and “inflammatory bowel disease,” consistent with the roles of airway muscle tone and CIH-induced pulmonary hypertension in OSA (Figure 3C). KEGG Pathway Analysis showed significant enrichment in pathways such as “NF-κB signaling,” “human cytomegalovirus infection,” and “apoptosis” (Figure 3D). Notably, GSEA further validated the “BioCarta Nfkb Pathway” as significantly enriched (NES = 1.730, *p*=0.014; Figure 3E), emphasising NF-κB signaling as a central mechanism in OSA.

### 3.5. Network Analysis of Key Targets in OSA

**Figure 4:**
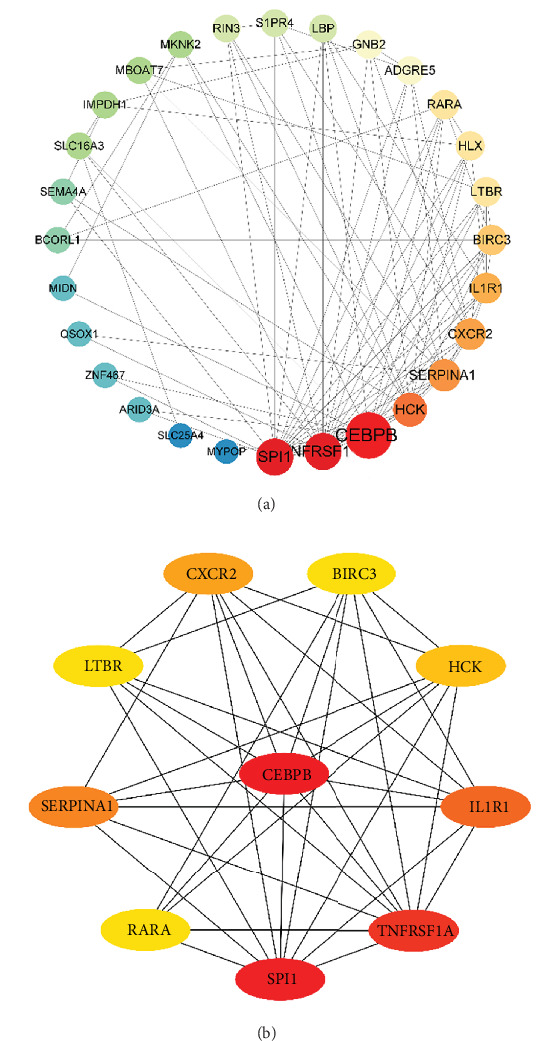
Protein–Protein Interaction Analysis of Key OSA Targets. (A) PPI network analysis of key OSA proteins, constructed after excluding six isolated nodes, highlighting the interconnected relationships among the core proteins. (B) Subnetwork analysis using the CytoHubba plugin, identifying hub proteins, such as CEBPB, SPI1, RARA, and TNFRSF11A, with CEBPB emerging as the central regulatory protein in OSA pathogenesis.

To explore the interactions among the 35 key targets in OSA, a PPI network was constructed using the STRING database. After excluding six isolated protein nodes, a comprehensive network of core gene interactions was generated (Figure 4A). Using the CytoHubba plugin in Cytoscape, a subnetwork was created to identify hub targets. Key genes, such as CEBPB, SPI1, TNFRSF1A, HCK, RARA, CXCR2, and LTBR, were identified, with CEBPB occupying the central position in the subnetwork (Figure 4B). These findings suggest that CEBPB may play a pivotal regulatory role in the pathogenesis of OSA.

### 3.6. Differential Analysis of Immune Cells in OSA

**Figure 5:**
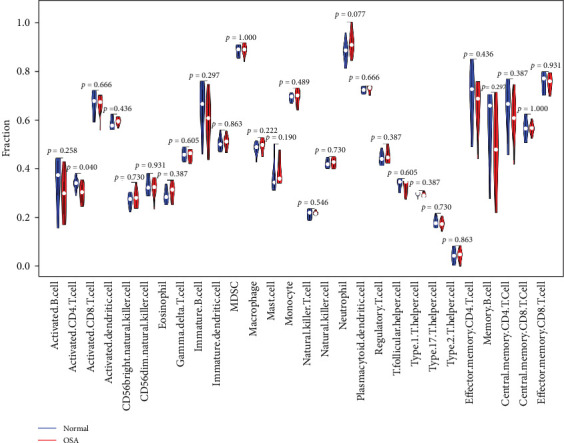
Analysis of differences in OSA immune cells.

Immune infiltration analysis revealed significant differences in CD4+ T cell levels between patients with OSA and controls (*p*  < 0.05). CD4+ T cell counts were significantly reduced in patients with OSA compared to controls (Figure 5). This result suggests that CD4+ T cells may play a crucial role in OSA pathogenesis, with their depletion potentially contributing to the immune dysregulation associated with the disease.

### 3.7. Single-Cell Analysis Reveals Immune Cell Alterations and NF-κB Pathway Activation in OSA

**Figure 6:**
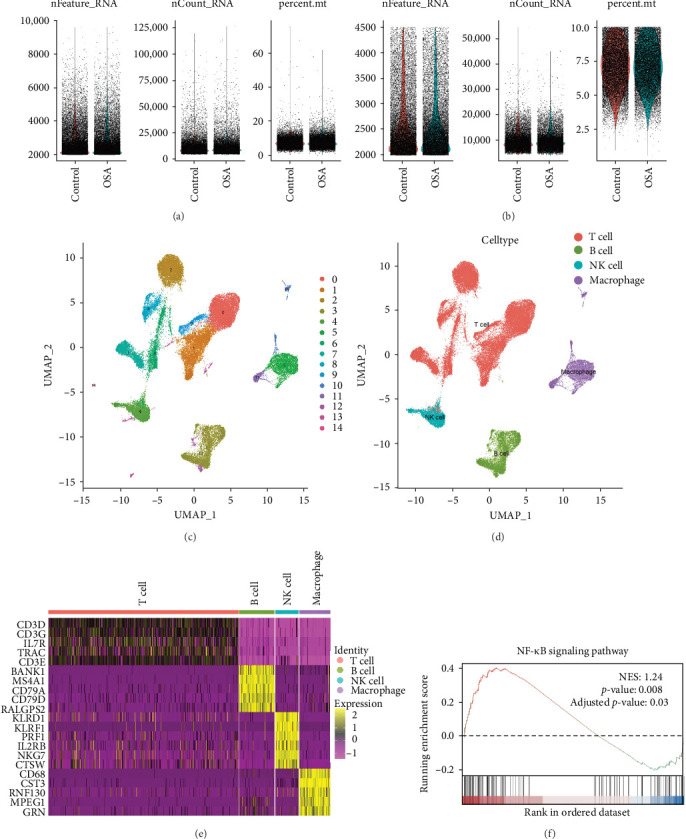
Single-cell sequencing reveals immune cell distribution and functional regulation in OSA patients. (A) Feature plot of single-cell RNA sequencing before quality control. (B) Feature plot of single-cell RNA sequencing after quality control. (C) UMAP plot showing the overall distribution of single cells. (D) UMAP immune cell classification, highlighting distinct immune cell types. (E) Heatmap of cell-type-specific gene expression. (F) GSEA analysis of the NF-κB pathway in T cells.nFeature_RNA: The number of genes detected in each cell; nCount_RNA: The total number of transcripts per cell (UMI count); percent.mt: The proportion of mitochondrial gene UMI counts in each cell.

To explore immune cell alterations in OSA, single-cell RNA sequencing (scRNA-seq) analysis was performed using the public dataset GSE214865. The dataset included 22 samples, comprising 11 PSG-confirmed patients with OSA (AHI > 5 events/h) and 11 controls. After quality control, 33,459 high-quality genes and 57,609 single cells were retained for analysis (Figure 6A,B). UMAP analysis identified 15 distinct cell clusters (Figure 6C), which were classified into four major groups—T cells, B cells, macrophages, and NK cells—based on canonical markers CD3D, CD19, CD68, and NCAM1 (Figure 6D). Differential expression and GSEA analysis revealed significant activation of the NF-κB signaling pathway (*p*  < 0.01) in T cells from patients with OSA (Figure 6E,F), suggesting that immune cell dysregulation in OSA is mediated, at least in part, by NF-κB pathway activation.

### 3.8. Single-Cell Analysis Reveals OSA May Activate the NF-κB Pathway to Affect T Cell Subpopulation Distribution

**Figure 7:**
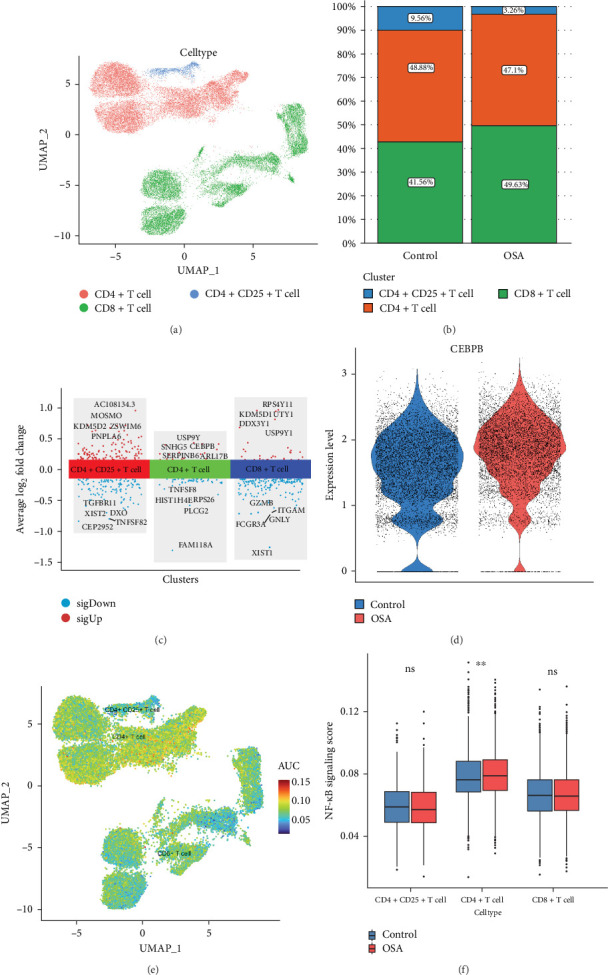
Impact of OSA on T cell subpopulations and potential mechanisms. (A) UMAP plot showing the classification of T cells. (B) Distribution of T cell subpopulations. (C) Volcano plot of differentially expressed genes in T cells. (D) Comparison of CEBPB gene expression levels in CD4+ T cells. (E) UMAP distribution of pathway activity scores across T cell subpopulations. (F) Comparison of NF-κB pathway activity in T cell subpopulations.

To further examine the effects of OSA on T cell subpopulations, specific markers (CD4, CD8, and CD25) were used to classify CD4+ T cells, CD8+ T cells, and regulatory T cells (Tregs; CD4+CD25+ T cells) (Figure 7A). Patients with OSA showed a significant reduction in the proportion of CD4+ T cells, particularly Tregs (Figure 7B). Notably, CEBPB expression was markedly upregulated in CD4+ T cells from patients with OSA (Figure 7C,D). Pathway activity analysis using the AUCell algorithm revealed heightened NF-κB signaling activity in CD4+ T cells compared to other T cell subpopulations (Figure 7E). Boxplots of pathway scores showed significantly elevated NF-κB activity in CD4+ T cells from patients with OSA (*p* < 0.01), while CD8+ T cells did not exhibit significant differences (*p* > 0.05) (Figure 7F). Analysis of cell clusters showed CD4+ T cells mainly in clusters 1, 3, and 5, while CD8+ T cells were mostly in clusters 2 and 4. OSA patients had a decreased proportion of CD4+ T cells across these clusters, whereas CD8+ T cell proportions were similar between groups. This suggests OSA mainly affects CD4+ T cell subsets via NF-κB pathway modulation. These findings highlight that OSA modulates T cell distribution and function, primarily through the NF-κB signaling pathway, with a notable impact on Tregs and CD4+ T cells.

### 3.9. Investigation of the Causal Relationship Between CEBPB Gene and NF-κB Signaling Pathway: A MR Analysis

**Figure 8:**
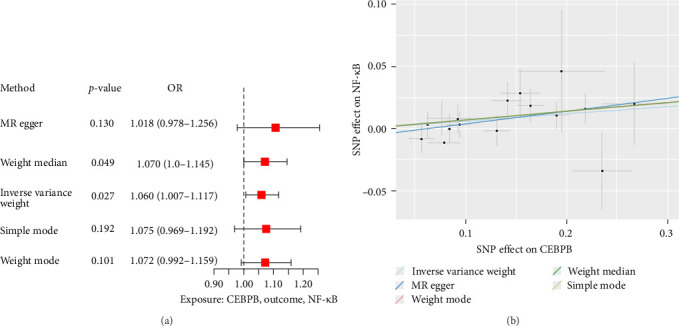
Mendelian randomization analysis between CEBPB and the NF-κB signaling pathway. (A) Forest plot of causal relationship between CEBPB gene and NF-κB pathway. (B) Scatter plot between CEBPB gene expression and NF-κB pathway.

To investigate the causal relationship between CEBPB and the NF-κB signaling pathway, MR analysis was conducted using the IVW method. Sixteen SNPs associated with CEBPB were analyzed. The IVW method identified a causal link between CEBPB and the NF-κB pathway, with an odds ratio (OR) of 1.060 (95% CI: 1.007–1.117, *p*=0.027; Figure 8A). The MR-Egger intercept test (*p*=0.369) indicated no significant pleiotropy, and Cochran's Q test revealed low heterogeneity (*Q* = 27.835, *p*=0.463). Scatter plot analysis further validated the causal association between CEBPB expression and NF-κB signaling activity (Figure 8B). These findings underscore the pivotal role of CEBPB and NF-κB signaling in OSA pathogenesis, offering potential therapeutic targets for modulating immune responses and mitigating disease progression.

## 4. Discussion

This study integrates transcriptomic data with PSG data to uncover key signaling pathways and core genes associated with OSA. In analyzing the clinical characteristics, no significant differences were observed in gender or BMI between patients with OSA and the normal population. However, critical indicators such as AHI, MSO2, LSO2, and ODI demonstrated significant variations, reflecting the degree of airway obstruction, alterations in sleep architecture, and the severity of CIH in patients with OSA. The degree of airway obstruction directly impacts AHI, while sleep fragmentation due to altered sleep architecture reduces sleep quality. Furthermore, CIH triggers stress responses in the cardiovascular and cerebrovascular systems, thereby elevating the risk of related diseases [11–13]. Analyzing these key indicators is essential for understanding the multifactorial pathophysiology of OSA. PCA of transcriptomic data revealed a clear separation between OSA and snoring populations along PC1 and PC2, suggesting differences in gene expression patterns. These differences likely relate to pathological mechanisms unique to OSA, including airway obstruction and CIH [14]. Additionally, the density distribution plot of gene expression further highlights the heterogeneity and specificity of gene expression profiles across samples.

DEGs represent variations in gene expression under distinct physiological or pathological states, such as disease versus healthy conditions. These genes often play critical roles in disease pathogenesis and progression and may serve as potential therapeutic targets [15]. Identifying DEGs in patients with OSA provides insight into the molecular mechanisms and pathways driving the disorder. WGCNA, a bioinformatics method for constructing gene coexpression networks, revealed the Palevioletred module as significantly correlated with OSA clinical parameters, including AHI, LSO2, MSO2, and ODI [16, 17]. By intersecting DEGs with genes in this module, core genes associated with OSA were identified. These findings enhance our understanding of the molecular underpinnings of OSA and provide potential avenues for future research into targeted therapies.

GO enrichment analysis identified 35 core genes associated with OSA clinical features, such as AHI. These genes are predominantly involved in BPs related to the response to lipopolysaccharides and bacterial-derived molecules, underscoring the pivotal role of inflammatory responses in OSA. Regarding CCs, the analysis highlighted peripheral structures on the cytoplasmic side of the cell membrane, including lipid rafts and microdomains, which are critical for intracellular signaling. In terms of MFs, the analysis revealed enrichment in histone deacetylase binding and cytokine binding. Lipopolysaccharides, a major component of Gram-negative bacterial cell walls, act as endotoxins capable of eliciting strong host immune responses [18]. Similarly, bacterial molecular patterns are recognized by the host immune system, triggering inflammation [19]. Membrane rafts and microdomains, specialized regions on the cell membrane, facilitate signal transduction crucial for cellular responses [20, 21]. Histone deacetylases, which remove acetyl groups from histone tails, regulate chromatin structure, and thereby influence gene expression and cellular functions [22].

This study identified CEBPB as a central hub gene within the core network. CEBPB is a transcription factor integral to various cellular processes, particularly inflammation, and immune responses. Notably, CEBPB is sensitive to hypoxic conditions and can be activated by hypoxia signals, subsequently regulating the expression of hypoxia-responsive genes [23]. In the context of OSA, recurrent apnoeic events induce CIH, which in turn upregulates CEBPB expression, amplifying inflammatory responses [24]. A complex interplay exists between CEBPB and the NF-κB signaling pathway. Recent evidence indicates that CEBPB activation is regulated by the NF-κB pathway, with both factors synergistically contributing to inflammation and immune cell dysregulation [25, 26]. scRNA-seq data further revealed elevated CEBPB expression in CD4+ T cells and concurrent activation of the NF-κB pathway. These findings suggest that inflammatory pathway activation influences immune cell proportions in OSA. Moreover, MR analysis confirmed a causal relationship between CEBPB expression and the NF-κB signaling pathway. The results indicate that increased CEBPB expression activates the NF-κB pathway.

KEGG and GSEA enrichment analysis results indicate that there is a close connection between the core genes of OSA and the NF-κB signaling pathway, which plays an important role in the development of OSA, especially in terms of inflammation. The enrichment of viral response pathways observed in KEGG and GSEA analyses may reflect chronic inflammation and persistent immune activation in OSA. Repeated hypoxia and sleep fragmentation in OSA can trigger immune dysregulation, resembling antiviral immune responses, which may contribute to the sustained inflammatory state and tissue damage associated with disease progression. The NF-κB pathway is a key route for cells to respond to external stimuli, involving BPs such as inflammation, immune responses, and cell proliferation [27]. External stimuli trigger receptor-mediated signal transduction, leading to the activation of NF-κB, which then translocates into the nucleus to regulate gene expression. NF-κB plays an important bridge role in converting extracellular signals into nuclear gene expression regulation [28, 29]. Apoptotic pathways, as a programed cell death process, are of significant importance to the life of cells. In the study of the pathogenesis of OSA, the interaction and regulatory mechanisms between the NF-κB pathway and apoptosis are particularly important, playing central roles in inflammation, cell damage, and repair [30, 31]. While this study has uncovered pivotal signaling pathways and core genes associated with OSA, several limitations warrant consideration. This study is limited by the relatively small sample size, which may affect the generalisability of the findings. Additionally, while transcriptomic and single-cell data suggest associations between core genes and the NF-κB pathway, functional validation through in vitro or in vivo experiments is still required. Last, MR analysis can infer causality statistically but cannot fully replace experimental confirmation of biological mechanisms.

## 5. Conclusion

This study highlights the pivotal role of the NF-κB signaling pathway in the pathogenesis of OSA, as revealed through integrative transcriptomic analysis, PSG data, and MR analysis. These findings deepen our understanding of OSA's molecular mechanisms and provide a solid basis for exploring targeted therapeutic strategies aimed at modulating the NF-κB pathway.

## Figures and Tables

**Table 1:** Comparison of clinical characteristics between OSA and primary snoring groups.

Parameter characteristics	Total (*n* = 18)	Control group (*n* = 9)	OSA group (*n* = 9)	Statistic	*p*-Value
Gender, *n* (%)	—	—	—	Fisher exact	1.000
Male	11	5 (55.56%)	6 (66.67%)	—	—
Female	7	4 (44.44%)	3 (33.33%)	—	—
Age, mean ± sd	61.67 ± 13.83	62.56 ± 10.37	60.78 ± 17.23	*t* = 0.27	0.794
BMI, mean ± sd	25.16 ± 4.64	24.44 ± 3.32	25.89 ± 5.79	*t* = −0.65	0.525
AHI, M (Q_1_, Q₃)	10.25 (4.55, 30.62)	4.50 (1.50, 4.90)	30.90 (29.60, 63.40)	*Z* = −3.53	<0.001
PRR, mean ± sd	26.72 ± 2.76	25.78 ± 2.91	27.67 ± 2.39	*t* = −1.50	0.152
MAHD, mean ± sd	22.28 ± 2.76	22.78 ± 3.56	21.78 ± 1.72	*t* = 0.76	0.459
LAD, mean ± sd	57.67 ± 11.99	51.56 ± 8.95	63.78 ± 11.88	*t* = −2.47	0.025
NAHE, mean ± sd	113.56 ± 133.00	19.78 ± 13.11	207.33 ± 132.79	*t* = −4.22	0.003
MSO_2_, mean ± sd	93.80 ± 1.97	94.74 ± 1.23	92.86 ± 2.19	*t* = 2.25	0.039
LSO_2_, mean ± sd	78.18 ± 12.56	86.35 ± 3.98	70.02 ± 13.02	*t* = 3.60	0.002
ODI, mean ± sd	21.02 ± 21.50	6.44 ± 5.14	35.60 ± 21.86	*t* = −3.89	0.004

*Note:* Gender; sex; age; female; male; *N*, number of participants. Parameters: t, *t*-test; Q_1_, 1st quartile; Q₃, 3rd quartile; PRR, peak respiratory rate; SD, standard deviation; *Z*, Mann–Whitney test.

Abbreviations: AHI, apnea–hypopnea index; BMI, body mass index; LAD, longest apnea and hypopnea duration; LSO_2_, lowest oxygen saturation; M, median; MAHD, mean apnea and hypopnea duration; MSO_2_, mean oxygen saturation; NAHEs, number of apnea and hypopnea events; ODI, oxygen desaturation index.

## Data Availability

The datasets generated and/or analyzed during the current study are available from the corresponding author upon reasonable request.
